# Blind Recognition Algorithm of Multi-Carrier Composite Modulation Signal Based on Multi-Dimensional Time-Frequency Superimposed Spectrum

**DOI:** 10.3390/s25134007

**Published:** 2025-06-27

**Authors:** Shoubin Wang, Huan Li, Xiaolong Zhang, Hao Jiang, Lei Shen

**Affiliations:** 1 The 36th Research Institute of China Electronics Technology Corporation, Jiaxing 314033, China; 222080115@hdu.edu.cn (S.W.); 222080244@hdu.edu.cn (H.J.); 2 College of Communication Engineering, Hangzhou Dianzi University, Hangzhou 310018, China; 222080174@hdu.edu.cn; 3 College of Computer Science and Engineering, Ocean University of China, Qingdao 266100, China; yufenfe2023@163.com

**Keywords:** multi-carrier composite modulation, blind recognition, MD-TFSS, DECA-ResNet18

## Abstract

The existing multi-carrier composite modulation recognition methods have failed to effectively integrate inner and outer modulation characteristics, thereby limiting the potential for improving recognition performance under low signal-to-noise ratio (SNR) conditions. To address this issue, this paper proposes a multi-carrier composite signal modulation recognition algorithm based on a multi-dimensional time-frequency superimposed spectrum (MD-TFSS) with integrated inner and outer features, which can recognize composite modulation signals in the set {BPSK-PM, QPSK-PM, BPSK-QPSK-PM, BPSK-BPSK-PM, QPSK-QPSK-PM}. The proposed method constructs a dual spectrum through multiplying an inner modulation spectrum and a squared spectrum, then combines the inner modulation dual spectrum with the outer modulation time-frequency diagram in dual-channel mode to form MD-TFSS features. Based on the MD-TFSS, a blind recognition algorithm is implemented using the dual-channel input ECA-ResNet18 (DECA-ResNet18) incorporating the ECA attention mechanism. The proposed algorithm first converts the complex features of multi-carrier composite modulation signals into visually interpretable image features (including the quantity and concentration of bright spots and lines) through the MD-TFSS, achieving intuitive representation of multiple modulation characteristics. Meanwhile, the dual-channel input mechanism enables collaborative expression of outer modulation time-frequency diagram and inner modulation dual spectrum features, ensuring tight integration of inner and outer characteristics while avoiding feature isolation issues in traditional multi-diagram concatenation methods. Secondly, the DECA-ResNet18 network dynamically allocates weights through an adaptive regulation mechanism based on input feature differences, autonomously adjusting channel attention levels to effectively capture complementary characteristics from both inner and outer modulation features, thereby enhancing recognition accuracy and generalization capability for multi-carrier composite modulation signals. Theoretical analysis and simulation results demonstrate that, compared with the existing methods that use isolated outer and inner features or conventional multi-feature diagram construction approaches, the proposed algorithm achieves superior recognition performance under low SNR conditions. Additionally, DECA-ResNet18 demonstrates enhanced recognition performance for multi-carrier composite modulated signals compared to the traditional ResNet18.

## 1. Introduction

Multi-carrier composite modulation signals are widely used in satellite communications, telemetry, tracking, ranging, and broadcasting systems. Compared to single-modulation communication signals, these complex modulation formats significantly increase signal recognition difficulty [1,2,3]. Communication signal recognition identifies modulation schemes automatically by jointly analyzing signal features (e.g., time and frequency domain features) under unknown channel conditions. It provides foundational support for subsequent in-depth analysis like signal demodulation and decoding, playing a critical role in electronic reconnaissance, information security, and battlefield situational awareness in complex electromagnetic environments. In non-cooperative communication scenarios lacking prior information and operating under low SNR environments, enhancing the recognition performance of multi-carrier composite modulated signals is more challenging [4]. Therefore, enhancing recognition capabilities for multi-carrier composite signals is crucial in non-cooperative communication systems [5]. The existing composite modulation recognition methods are broadly categorized into feature extraction-based algorithms and deep learning-based algorithms.

In the realm of feature extraction-based recognition algorithms, References [6,7] introduce a method that directly extracts the time-frequency domain features of the outer modulation to recognize signals. This approach allows for feature extraction and signal recognition without removing the main carrier. However, the selected features are relatively simple and only utilize outer modulation features, limiting the types of signals that can be distinguished. Reference [8] constructs signal features based on instantaneous histogram features and cyclic spectra, which also rely solely on outer modulation features, resulting in low recognition rates under low SNR conditions. Reference [9] employs the analytic orthogonal transformation method to separate the main carrier and subcarriers of the composite modulated signal and completes signal recognition by extracting the high-order spectrum features of the subcarrier signal. Reference [10] first demodulates the composite modulated signal and then extracts the instantaneous phase of the inner modulation signal, leveraging the relationship between the instantaneous phase and time domain data of the inner modulation signal for recognition. Both of these methods only utilize inner modulation features and are easily constrained by the preprocessing performance. Reference [11] uses the fourth-order cumulants of the outer modulation signal and the squared spectrum features of the inner modulation signal, employing a decision tree classifier to achieve modulation recognition of MPSK-PM signals, but still independently uses inner and outer modulation features. The aforementioned methods can be categorized into three types: the first relies solely on outer modulation features; the second uses only inner modulation features; and the third employs inner and outer features independently. These approaches fail to tightly integrate the inner and outer modulation characteristics of composite modulated signals, thereby limiting their potential for improved recognition performance under low SNR conditions.

In terms of deep learning-based recognition algorithms, Reference [12] focuses on outer modulation features by extracting frequency-domain features and using a BP neural network for classification. Reference [13] utilizes outer modulation spectrum and squared spectrum peak features for recognition, while Reference [14] employs the frequency-domain data of outer modulation and applies compressive sensing for feature dimensionality reduction, directly recognizing the compressed data. These three methods only use outer modulation features, resulting in a limited applicable signal set and a drop in recognition rate under multi-carrier conditions. Reference [15] proposes a hierarchical recognition method for composite signals based on outer modulation spectrum features, inner modulation frequency-domain features, and high-order spectrum features, but the inner and outer modulation features are still used independently, leading to low recognition rates under low SNR conditions. Reference [16] constructs a feature vector based on the instantaneous amplitude, spectral line features, spectrum features of the outer modulation signal, as well as the information component features of the inner modulation signal, using a backpropagation neural network classifier for recognition. This method simply combines the inner and outer features, so it is not tight enough, and still cannot recognize multi-carrier signals. Reference [17] employs a method of directly concatenating multiple feature maps for recognition, but when applied to composite modulation signals, the inner and outer features remain independently distributed. It can be observed that deep learning-based composite modulated signal recognition methods exhibit three analogous categories to feature extraction-based approaches. The utilization of inner and outer modulation features also remains at the stage of independent utilization or simplistic combination, likewise constraining the enhancement of recognition performance.

This paper proposes a blind modulation recognition algorithm for multi-carrier composite modulated signals, integrating MD-TFSS analysis with the DECA-ResNet18 network for feature extraction and classification. The main contributions are summarized as follows.

- This paper introduces the construction of the MD-TFSS by integrating the outer modulation time-frequency spectrum and the inner modulation dual spectrum. The MD-TFSS achieves deep fusion of the inner and outer modulation characteristics of multi-carrier composite modulated signals, resulting in a synergistic representation of their combined modulation features.
- This paper proposes the DECA-ResNet18 network, which effectively captures the complementarity of inner and outer modulation features across different channels, thereby enhancing the recognition performance of the MD-TFSS.
- This paper compares the performance improvements of MDT-FSS against methods that use isolated outer features, standalone inner features, or conventional multi-feature fusion approaches. It further demonstrates the superiority of DECA-ResNet18 over the traditional ResNet in recognizing the dual-channel MD-TFSS.
- The remainder of the paper is organized as follows: Section 2 introduces the multi-carrier composite modulated signal model. Section 3 details the construction methodology of the multi-dimensional time-frequency composite spectrum. Section 4 introduces the architecture and principles of the DECA-ResNet18 network. Section 5 presents the relevant simulation results. Section 6 elucidates the conclusions.

## 2. Multi-Carrier Composite Modulated Signal Model

Multi-carrier composite modulated signals enhance the spectral efficiency, transmission rate, and quality of communication systems by superimposing multiple carriers. The composite modulation signals studied in this paper include BPSK-PM, QPSK-PM, BPSK-QPSK-PM, BPSK-BPSK-PM, and QPSK-QPSK-PM. These types of signals are widely used in telemetry, tracking, and command systems (TT&C) [18].

Assuming that the modulation of the composite signal includes N subcarriers, for example, the value of N is 1 for BPSK-PM and QPSK-PM signals, whereas it is 2 for BPSK-QPSK-PM, BPSK-BPSK-PM, and QPSK-QPSK-PM signals. The inner modulation signals, represented by BPSK and QPSK signals as the subcarriers, can be expressed as follows:

(1) $$s_{i}(t)=a_{i}(t)\cos(2\pi f_{i}(t)+\varphi_{i,0})-b_{i}(t)\sin(2\pi f_{i}(t)+\varphi_{i,0}),$$

where $f_{i}(t)$ and $\varphi_{i,0}(i\in \{1,2,\ldots ,N\})$ represent the frequency and initial phase of the *i*-th subcarrier, respectively. For BPSK signals, $a_{i}(t)$ takes values of ±1, and $b_{i}(t)$ is 0. For QPSK signals, both $a_{i}(t)$ and $b_{i}(t)$ can take values of ±1. The outer modulation type is PM modulation for all. The PM signal can be expressed as follows:

(2) $$s_{PM}(t)=C_{p}\cos\left[2\pi f_{p}(t)+K_{p}\cdot m(t)+\Phi_{0}\right].$$

In the formula, $C_{p}$, $f_{p}$, $\Phi_{0}$ represent the amplitude of the outer PM modulation signal, the carrier frequency, and the initial carrier phase, respectively; $K_{p}$ is the phase modulation index, with a normal range of $0.2\leq K_{p}\leq 1.4$; and m(t) is the baseband signal. Substituting the accumulated $s_{i}(t)$ in Equation (1) into Equation (2) as the baseband signal m(t), the composite modulation signal S(t) for band transmission composed of N subcarriers can be represented as follows:

(3) $$S_{CM}(t)=C_{p}\cos\left[2\pi f_{p}(t)+K_{p}\sum_{i=1}^{N}s_{i}(t)+\Phi_{0}\right].$$

Taking the BPSK-QPSK-PM signal as an example, in its mathematical model with N set to 2, the parameters $a_{1}(t)$, $a_{2}(t)$, and $b_{2}(t)$ take values of ±1, while $b_{1}(t)$ takes 0.

## 3. The Joint Feature Extraction of Inner and Outer Modulation Based on the MD-TFSS

The MD-TFSS transforms complex multi-carrier composite modulation features into visually interpretable image characteristics (including the quantity and concentration of bright spots and lines), enabling intuitive modulation representation. Simultaneously, its dual-channel fusion mechanism integrates outer modulation time-frequency diagrams with the inner modulation dual spectrum, ensuring cohesive feature fusion while eliminating the isolation issues inherent in traditional multi-diagram concatenation methods.

### 3.1. Feature Extraction of Inner Modulation Based on Dual Spectrum

The dual spectrum serves as one of the input channels of the MD-TFSS, characterized by the inner modulation features of multi-carrier composite modulation signals. The inner modulation data are obtained by demodulating the outer modulation from the original signal data. Specifically, a Costas loop is employed for carrier synchronization of the outer phase-modulated signal [19], with an arctangent phase detector utilized as the phase discriminator. The specific implementation process is detailed below.

The squared spectrum and quadrupled spectrum of BPSK and QPSK exhibit impulse spectral line characteristics [20]. The dual spectrum is obtained by multiplying the spectrum $X(f_{1})$ and the squared spectrum $R_{2}(f_{2})$ of the inner modulation signal, as shown in Equation (4):

(4) $$XR_{2}(f_{1},f_{2})=\left|X(f_{1})R_{2}(f_{2})\right|.$$

The spectrum of the multi-carrier inner modulation signal $K_{p}\sum_{i=1}^{N}s_{i}(t)$ can be expressed as follows:

(5) $$X(f_{1})=\int_{-\infty }^{\infty }\left[K_{p}\sum_{i=1}^{N}s_{i}(t)\right]e^{-j2\pi ft}dt.$$

Its squared spectrum can be expressed as follows:

(6) $$R_{2}(f)=\int_{-\infty }^{\infty }E\left[{\left(K_{p}\sum_{i=1}^{N}s_{i}(t)\right)}^{2}\right]e^{-j2\pi ft}dt.$$

According to Formula (6), the squared spectrum of BPSK exhibits impulsive spectral lines, whereas that of QPSK does not. Consequently, in Formula (4), BPSK’s spectral lines manifest as concentrated bright spots, while those of QPSK appear as dispersed spots.

**Figure 1:**
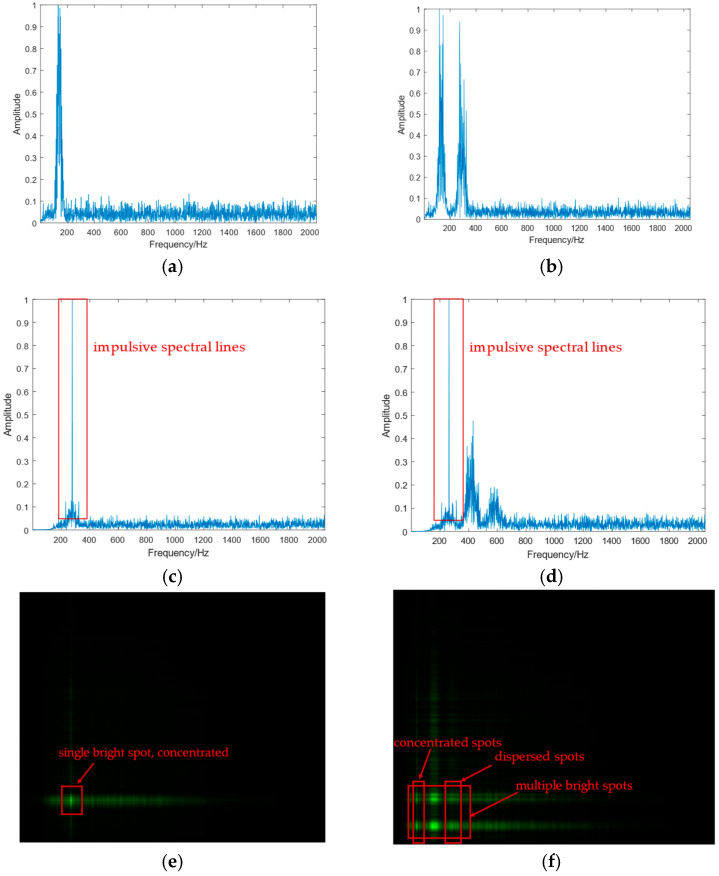
Inner modulation feature map. (**a**) BPSK-PM inner modulation spectrum. (**b**) BPSK-QPSK-PM inner modulation spectrum. (**c**) BPSK-PM inner modulation square spectrum. (**d**) BPSK-QPSK-PM inner modulation square spectrum. (**e**) BPSK-PM inner modulation dual spectrum. (**f**) BPSK-QPSK-PM inner modulation dual spectrum.

Taking BPSK-PM and BPSK-QPSK-PM as examples, their inner modulation feature diagrams are shown in Figure 1.

As shown in Figure 1a,b, the distinction between single-carrier and multi-carrier signals can be clearly observed in their spectrograms. Analysis of the inner modulation squared spectrum diagrams in Figure 1c,d reveals distinct differences between inner-modulated BPSK and QPSK sub-carrier signals: the BPSK signal exhibits impulsive spectral lines in its squared spectrum, while the QPSK signal lacks such features. The dual spectrum (Figure 1e,f) encodes the inner modulation characteristics of composite signals through the number and spatial concentration of bright spots and lines, where multi-carrier signals display multiple bright spots, single-carrier signals show a single bright spot, inner-modulated BPSK signals exhibit highly concentrated spots and lines, and inner-modulated QPSK signals present dispersed spots and lines.

### 3.2. Construction of MD-TFSS

The MD-TFSS is constructed from the outer modulation time-frequency spectrum and the inner modulation bilinear spectrum. The time-frequency plot of the outer modulation signal can be obtained through the Short-Time Fourier Transform (STFT):

(7) $$T_{s}(t,f)=\int_{-\infty }^{\infty }S(u)\cdot w(t-u)e^{-j2\pi fu}du$$

The Hanning window function is applied to the STFT to obtain the time-frequency distribution of the signal. The amplitude values extracted from the STFT results are normalized to construct the time-frequency diagram.

**Figure 2:**
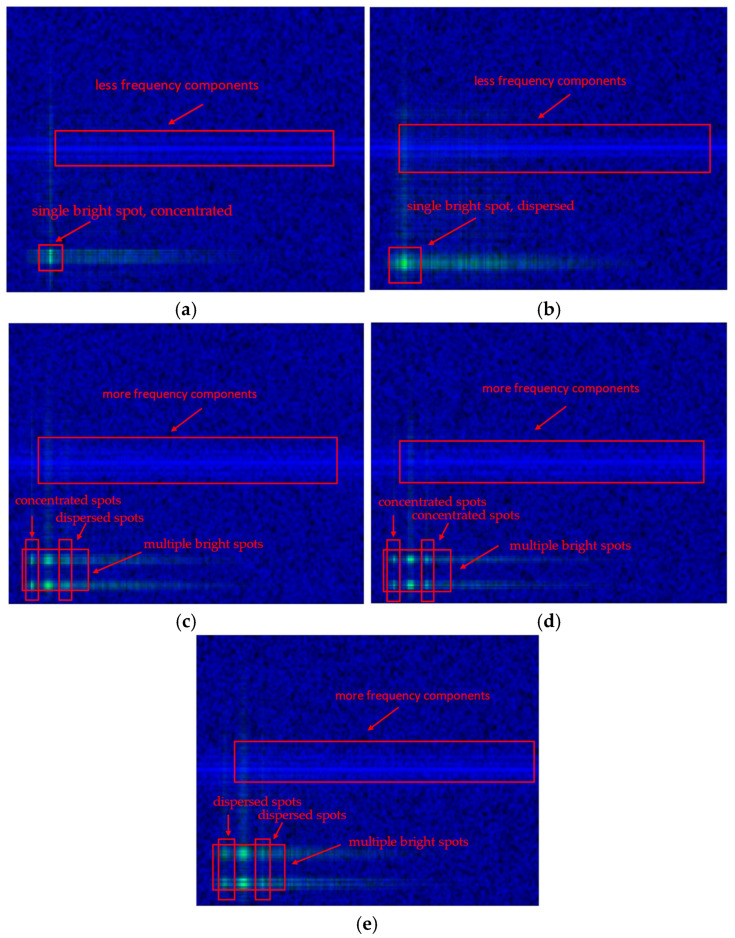
Multi-dimensional time-frequency superimposed spectrum. (**a**) BPSK-PM. (**b**) QPSK-PM. (**c**) BPSK-QPSK-PM. (**d**) BPSK-BPSK-PM. (**e**) QPSK-QPSK-PM.

The outer modulation time-frequency diagram and the inner modulation dual spectrum (obtained by multiplying the inner modulation spectrogram and squared spectrum) are input into the blue and green channels of an RGB image, respectively, to generate a joint inner–outer modulation feature map, with the red channel left empty. The resulting MD-TFSS is illustrated in Figure 2.

As shown in Figure 2, the outer modulation time-frequency diagrams of single-carrier and multi-carrier composite modulation signals exhibit distinct differences: the former has significantly fewer frequency components than the latter. Additionally, the time-frequency spectrum enables the precise capture of outer modulation time-frequency characteristics in multi-carrier composite signals, facilitating feature extraction for subsequent neural networks.

The inner modulation spectrum provides frequency distribution information, while the squared spectrum reveals nonlinear interactions between frequency components. Green bright spots in the diagram represent intersections of the inner modulation spectrum and the squared spectrum. Single-carrier and multi-carrier signals are distinguishable by the number of bright spots. Meanwhile, BPSK and QPSK signals are differentiated by the concentration of bright spots and their spectral lines: BPSK exhibits highly concentrated bright spots due to impulsive spectral lines in its squared spectrum, whereas QPSK shows dispersed bright spots due to the absence of such lines.

Combined with the frequency component count from the outer modulation time-frequency spectrum, the MD-TFSS enhances discriminability between signal types in the time-frequency domain, capturing complex inner modulation characteristics and effectively integrating inner and outer modulation features to improve deep learning model performance.

## 4. Blind Recognition of Multi-Carrier Composite Signals Based on DECA-ResNet18

DECA-ResNet18 is an enhanced ResNet18-based architecture tailored for MD-TFSS image classification. The network processes MD-TFSS images through an initial feature extraction stage comprising convolutional, normalization, activation, and pooling operations, generating low-level feature representations. Its core structure integrates four sequential ECA blocks, which refine feature discriminability by modeling inter-channel dependencies. The network outputs a probability distribution over the modulation classes, with the final prediction assigned to the class exhibiting the maximum posterior probability.

The traditional ResNet18 model primarily relies on convolutional operations for local feature extraction. It struggles to effectively model the interdependencies between features across channels, often leading to overemphasis on specific channels and neglect of the overall composite feature structure. In this work, the distinct color channels of the input image carry heterogeneous and complementary features: outer modulation time-frequency features and inner modulation dual spectrum features. The proposed DECA-ResNet18 model incorporates an ECA channel attention mechanism within each residual module. This effectively enhances the model’s ability to model relationships between channels, significantly boosting its recognition performance for dual-channel RGB MD-TFSS images.

**Figure 3:**
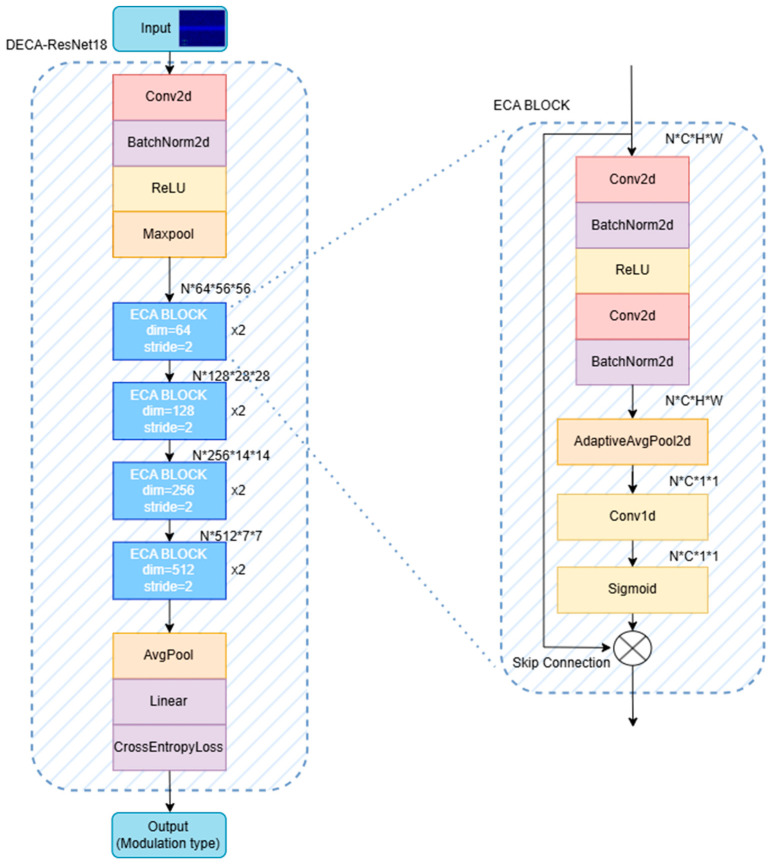
Structure diagram of DECA-ResNet18 network.

As shown in Figure 3, the input to the DECA-ResNet18 network is the joint inner–outer modulation feature map (224 × 224 pixels), fed as dual channels. The input first passes through a 7 × 7 convolutional layer (stride = 2, padding = 3) to extract primary edge and texture features, halving the feature map size. This is followed by BatchNorm2d for activation normalization, ReLU for nonlinear enhancement, and a MaxPool layer to further reduce dimensionality while retaining salient features.

Layers 1–4 stack multiple ECA residual blocks. The ECA module performs fine-grained modeling of channel-wise features after the output of each residual block. It first applies Adaptive Average Pooling to compress the two-dimensional feature map of each channel across the spatial dimensions, yielding a scalar representation that reflects the global response intensity of that channel across the entire feature map. This step enables the network to assess the importance of each channel holistically, laying the foundation for subsequent attention allocation.

Subsequently, the ECA module employs a 1D convolution that slides along the channel dimension to model the relationships between each channel and its neighboring channels. This design ensures that the attention coefficient for each channel is not dependent only on its own statistical features, but is also influenced by the synergistic effects of its local channel neighborhood. This approach to local channel interaction modeling enhances the network’s sensitivity to “cross-channel complementary features” in RGB images. This is particularly beneficial for achieving balanced attention when inner and outer modulation features are superimposed, reducing the model’s tendency to bias towards either modulation component.

Immediately following this, the convolutional output passes through a Sigmoid activation function, mapping the weights to the 0–1 interval to serve as importance coefficients for each channel. These coefficients are then multiplied element-wise with the original feature maps. This process explicitly enhances key modulation features while suppressing redundant information. Crucially, it achieves this without introducing additional structural complexity, thereby strengthening the model’s ability to focus on discriminative regions of modulation signals. Especially when identifying typical spectral features like bright lines or texture discontinuities in images, the ECA module guides the network to concentrate more effectively on discriminative regions.

Ultimately, this design—combining the channel recalibration mechanism with skip connection structures—not only enhances the deep features’ ability to fuse and express inner and outer modulation information, but also ensures the effective propagation of shallow features. This forms a cross-level, multi-scale feature enhancement pathway. Overall, the ECA module endows the DECA-ResNet18 architecture with significantly stronger selective attention capability and higher discriminative power when faced with complex, multi-source modulated feature inputs. This effectively overcomes the problem of channel attention imbalance that traditional ResNet18 structures exhibit when processing MD-TFSS images.

After processing through all ECA residual blocks, a global average pooling layer (AvgPool) compresses the spatial dimensions to retain essential global features. A fully connected layer (Linear) maps these features to the classification space, with the final results output via Softmax. This architecture enables the effective extraction and dynamic fusion of inner–outer modulation features, significantly improving generalization and recognition accuracy for multi-carrier composite modulation signals.

The DECA-ResNet18 network effectively divides its attention between dual-channel input features through an adaptive regulation mechanism, dynamically adjusting inter-channel weight allocations based on input feature differences. The integrated features are then used for network training, enhancing complementary characteristics across channels. This mechanism improves model accuracy and generalization during composite modulation signal recognition tasks while boosting adaptability in complex scenarios such as blind recognition of multi-carrier composite modulation signals. Additionally, compared to traditional attention mechanisms, the ECA module achieves this with lower computational complexity and minimal overhead.

## 5. Simulation Results and Analysis

### 5.1. Data Collection

The experimental signal set {BPSK-PM, QPSK-PM, BPSK-QPSK-PM, BPSK-BPSK-PM, QPSK-QPSK-PM} consists of theoretical signals generated in MATLAB 2024a. The specific parameter settings are listed in Table 1. The Inner Modulation Symbol Rate, Sub-carrier 1 Frequency, and Sub-carrier 2 Frequency are uniformly distributed within the ranges specified in the table. Sub-carrier 1 and sub-carrier 2 use different carrier frequency settings to prevent the subcarrier signals from interfering with each other. Given the Gaussian noise-dominated channel characteristics of the TT&C systems targeted by this research, AWGN channel models were employed during simulations for fidelity. For SNR levels ranging from −8 dB to 10 dB with 2 dB intervals, 800 samples per SNR level per signal type were generated, totaling 40,000 samples, with 32,000 for training and 8000 for testing.

### 5.2. Experimental Environment and Parameter Settings

The experiments utilized an NVIDIA GeForce RTX 3090 GPU with the PyTorch 2.6.0 framework. Inputs included dual-channel feature maps with a resolution of 224 × 224 pixels and traditional concatenated feature maps. The learning rate was fixed at 0.0005, the batch size was 16, and training lasted 40 epochs. The DECA-ResNet18 model was trained and tested on three types of feature maps and traditional concatenated feature maps.

### 5.3. Experimental Results Analysis

**Figure 4:**
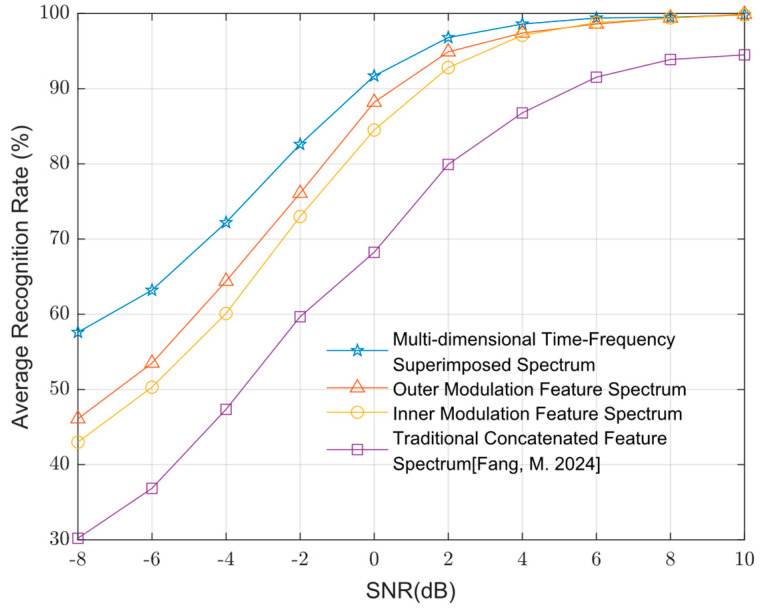
Average recognition rates of different feature maps at various SNRs [17].

Figure 4 presents the experimental statistical results of the average recognition performance for the signal set using four types of feature maps as model inputs across various SNR levels. The recognition result is for multiple classes. The average recognition rate shown in Figure 4 is the average recognition rate of five modulation types. As shown in Figure 4, the joint inner–outer modulation features achieved an approximately 8% higher average recognition performance compared to using inner modulation features alone and 6% higher than using outer modulation features alone. This indicates that the joint features enable the model to better learn the characteristics of composite modulation signals, significantly improving recognition performance for composite signals under low SNR conditions. The traditional concatenated feature maps, which linearly combine outer modulation time-frequency diagrams, inner modulation spectra, and inner modulation squared spectra as described in Reference [17], yielded the lowest recognition performance.

**Figure 5:**
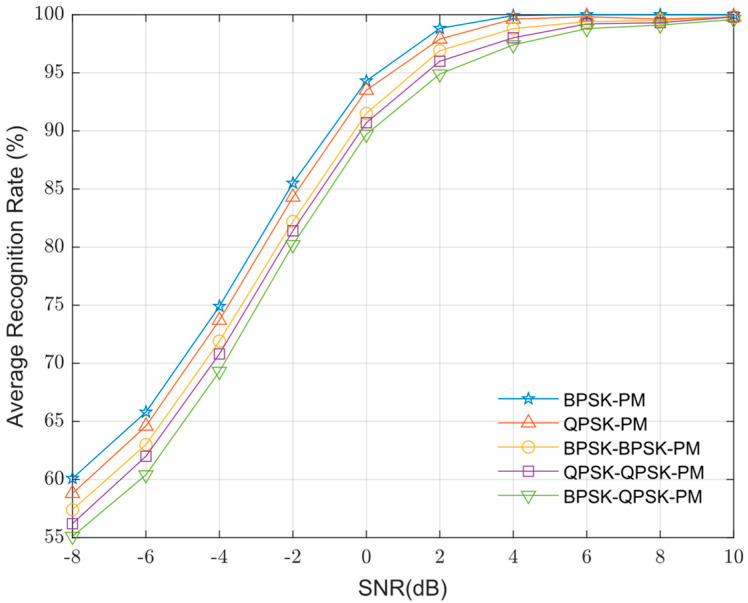
Individual average recognition rates of five signals at various SNRs.

**Figure 6:**
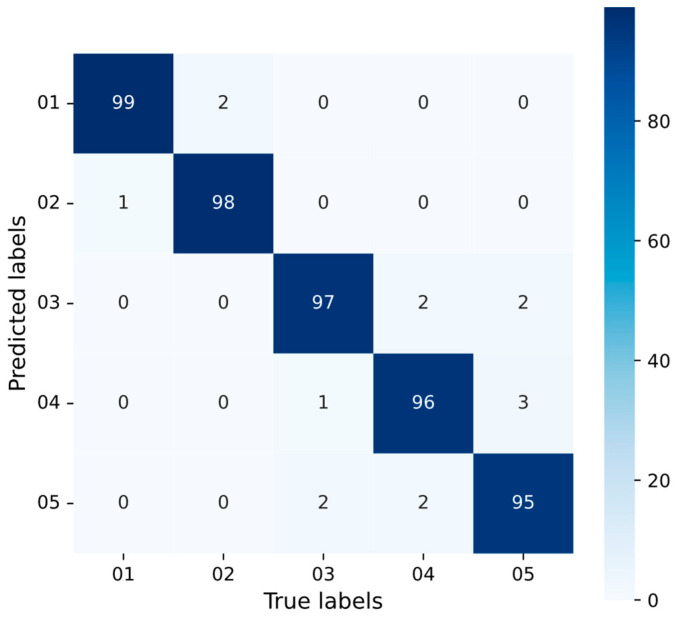
Confusion matrix of proposed method at 2 dB SNR.

Figure 5 presents the individual average recognition rates of the five modulation types across various SNRs. Figure 6 presents the confusion matrix for five modulation types (2 dB SNR): 01 (BPSK-PM), 02 (QPSK-PM), 03 (BPSK-BPSK-PM), 04 (QPSK-QPSK-PM), and 05 (BPSK-QPSK-PM). As observed in Figure 2, Figure 5 and Figure 6, the MD-TFSS features of BPSK-PM exhibit the simplest and clearest structure, characterized by fewer time-frequency components in the outer modulation feature map and inner modulation features containing only a single concentrated bright spot. These characteristics enable it to achieve the highest recognition rate. Conversely, the MD-TFSS features of BPSK-QPSK-PM display the highest complexity, featuring numerous time-frequency components in the outer modulation feature map and inner modulation characteristics that show multiple concentrated or dispersed bright spots and lines. This structural complexity corresponds to its low recognition rate. In addition, BPSK-PM and QPSK-PM exhibit similar recognition rates due to comparable MD-TFSS feature complexity, as do BPSK-BPSK-PM and QPSK-QPSK-PM.

**Figure 7:**
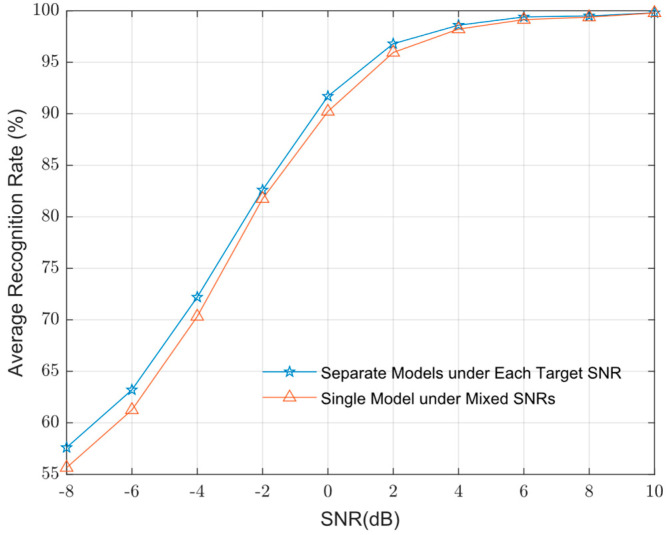
Performance of models trained under each target SNR and mixed SNRs.

In this study, we train separate models for each target SNR. However, the training data used in practical TT&C scenarios often contain mixed SNRs. To evaluate performance under these realistic conditions, we also train a single model using mixed-SNR data. Its simulation results are shown in Figure 7.

In Figure 7, the recognition performance of the model trained on mixed-SNR data is slightly lower than that of the models trained on individual SNRs. Nevertheless, it still maintains a high recognition rate. These results validate the performance of the proposed algorithm in practical scenarios.

**Figure 8:**
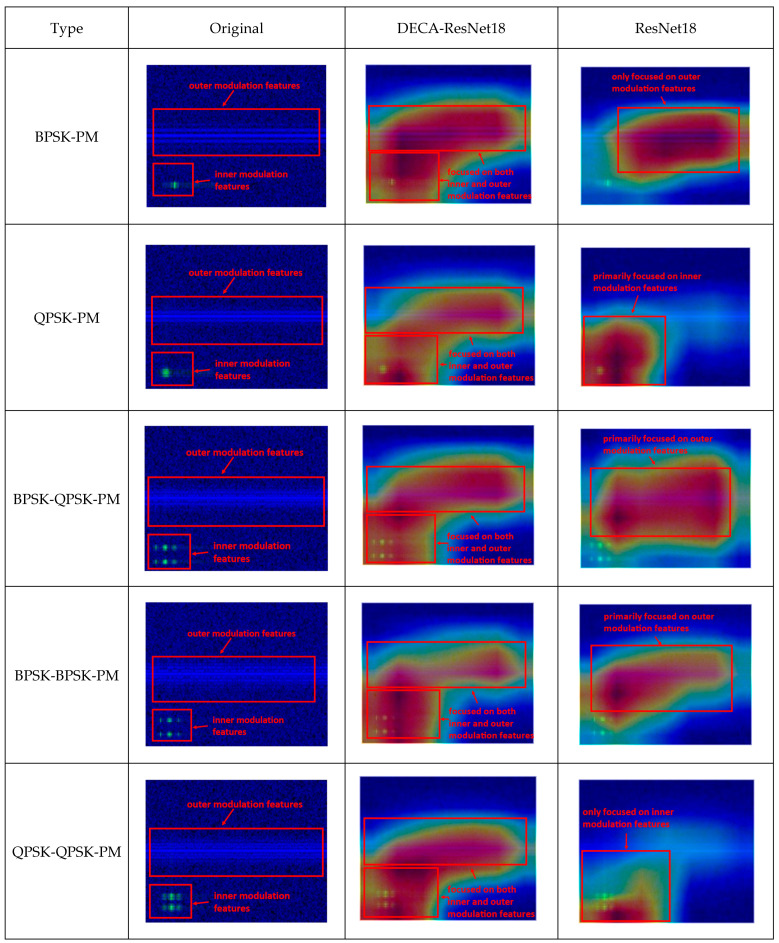
Visualized heat maps.

The DECA-ResNet18 model described in this paper is enhanced based on the traditional ResNet18 [21]. We visualized the features extracted by the proposed model and the traditional ResNet18 model using heatmaps. In these heatmaps, red regions represent the composite modulation features learned by the models, indicating areas of focused attention during training. Figure 8 shows the feature visualization heatmaps for both network models.

As shown in Figure 8 (where inner and outer modulation feature distributions are marked in the “original” column), the standard ResNet18 over-focuses on either central inner or lower-left outer modulation features, consistent with Section 4. DECA-ResNet18 addresses this by adaptively balancing its attention between dual-channel inputs. Its dynamic weighting mechanism enables complementary feature integration, boosting accuracy and generalization during the blind recognition of multi-carrier composite modulated signals.

**Figure 9:**
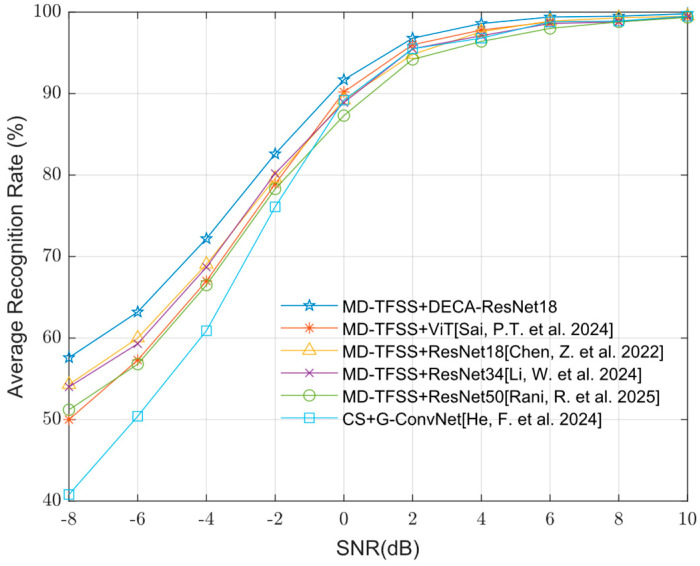
Average recognition rates of different network models under different SNRs [14,21,22,23,24].

Figure 9 compares the average recognition performance of five modulation signals using AWGN channels across the SNR range from −8 dB to 10 dB, with all other parameters consistent with previous settings. The performance of the following models is evaluated: DECA-ResNet18, Vision Transformer (ViT) [21], ResNet18 [22], ResNet34 [23], ResNet50 [24], and the algorithm proposed in Reference [14].

As shown in Figure 9, when the ECA attention mechanism is incorporated into the ResNet18 network, the average recognition rate improves by about 3% under low SNR conditions. Moreover, the algorithm in this paper based on the MD-TFSS, and DECA-ResNet18 demonstrates superior performance compared to the other methods.

## 6. Conclusions

This study addresses the recognition of composite modulation signals {BPSK-PM, QPSK-PM, BPSK-QPSK-PM, BPSK-BPSK-PM, QPSK-QPSK-PM} by proposing a recognition algorithm based on the MD-TFSS. A dual spectrum joint feature map is constructed by integrating inner modulation spectra and squared spectra, while outer modulation features are extracted from time-frequency diagrams. The inner and outer modulation features are then combined in dual-channel form to generate the MD-TFSS. Finally, the DECA-ResNet18 model with the ECA attention mechanism is employed to learn these features, significantly improving the recognition rate for multi-carrier composite modulation signals under low SNR conditions.

## Figures and Tables

**Table 1:** Specific parameter settings.

Parameters	Specific Numerical Values
Main Carrier Frequency	240 kHz
Inner Modulation Symbol Rate	10–20 kBaud/s
Sub-carrier 1 Frequency	20–40 kHz
Sub-carrier 2 Frequency	40–80 kHz
Sampling Rate	960 kHz

## Data Availability

The original contributions presented in this study are included in the article. Further inquiries can be directed to the corresponding author.
